# New natural products identified by combined genomics-metabolomics profiling of marine *Streptomyces* sp. MP131-18

**DOI:** 10.1038/srep42382

**Published:** 2017-02-10

**Authors:** Constanze Paulus, Yuriy Rebets, Bogdan Tokovenko, Suvd Nadmid, Larisa P. Terekhova, Maksym Myronovskyi, Sergey B. Zotchev, Christian Rückert, Simone Braig, Stefan Zahler, Jörn Kalinowski, Andriy Luzhetskyy

**Affiliations:** 1 Helmholtz-Institute for Pharmaceutical Research Saarland, Actinobacteria Metabolic Engineering Group, Saarbrücken, Germany; 2 Gause Institute of New Antibiotics, Russian Academy of Medical Sciences, Moscow, Russia; 3 Department of Biotechnology, Norwegian University of Science and Technology, Trondheim, Norway; 4 Department of Pharmacognosy, University of Vienna, Vienna, Austria; 5 Center for Biotechnology, Bielefeld University, Bielefeld, Germany; 6 Department of Pharmacy - Center for Drug Research, University of Munich, Munich, Germany; 7 Universität des Saarlandes, Pharmaceutical Biotechnology, Saarbrücken, Germany

## Abstract

Marine actinobacteria are drawing more and more attention as a promising source of new natural products. Here we report isolation, genome sequencing and metabolic profiling of new strain *Streptomyces* sp. MP131-18 isolated from marine sediment sample collected in the Trondheim Fjord, Norway. The 16S rRNA and multilocus phylogenetic analysis showed that MP131-18 belongs to the genus *Streptomyces*. The genome of MP131-18 isolate was sequenced, and 36 gene clusters involved in the biosynthesis of 18 different types of secondary metabolites were predicted using antiSMASH analysis. The combined genomics-metabolics profiling of the strain led to the identification of several new biologically active compounds. As a result, the family of bisindole pyrroles spiroindimicins was extended with two new members, spiroindimicins E and F. Furthermore, prediction of the biosynthetic pathway for unusual α-pyrone lagunapyrone isolated from MP131-18 resulted in foresight and identification of two new compounds of this family – lagunapyrones D and E. The diversity of identified and predicted compounds from *Streptomyces* sp. MP131-18 demonstrates that marine-derived actinomycetes are not only a promising source of new natural products, but also represent a valuable pool of genes for combinatorial biosynthesis of secondary metabolites.

The discovery of new antibiotics remains one of the most important tasks of modern biotechnology due to the rapid emergence of antibiotic resistance among pathogenic bacteria^1^. The latter leads to an increasing number of untreatable or poorly treatable bacterial infections, which can potentially become one of the leading causes of mortality^2^. This makes the search for new antimicrobial compounds of vital importance for modern medicine.

Two thirds of all antibiotics originate from biological sources or are the semi-synthetic derivatives of biologically produced natural compounds^3^. Actinomycete bacteria, in particular those of the genus *Streptomyces*, are one of the most promising biological sources of new natural products, and will remain so at least for the near future^4^. Due to the diverse secondary metabolism these bacteria accumulate a large number of compounds, which may become drug leads for the development of antibacterials, antivirals, immunosuppressants, antifungals, insecticides, and antitumorals. The intensive exploration of terrestrial actinomycetes in 1950–70 s has led to frequent re-discovery of bioactive compounds, thus drawing interest to new ecological niches, which may become sources of new actinomycetes^5^. Given that oceans cover more than 70% of the Earth’s surface and host approximately 87% of global biodiversity, they appear largely underexplored in terms of discovery of new microorganisms, including actinomycetes. Taking into consideration unprecedented diversity of marine organisms and comparatively little work done so far, the reported identification of more than 20.000 new marine natural products is astounding^6^. Many of these compounds are produced by marine actinomycetes isolated from the deep sea sediments, coral reefs, marine invertebrates, and plants^7^,^8^. The taxonomic diversity of these actinomycetes ranges from typical representatives of *Streptomyces* to more exotic and rare *Dietzia, Rhodococcus, Salinispora, Marinophilus, Solwaraspora, Salinibacterium, Aeromicrobium, Williamsia* and *Verrucosispora* species, increasing the chances for the discovery of new bioactive natural products^8^. Several compounds produced by marine actinomycetes have a strong potential to be developed into pharmaceutical drugs. Diazepinomicin, a dibenzodiazepine alkaloid from a marine *Micromonospora* strain, possessed antibacterial and antitumor activities, and had been in phase II clinical trials for the treatment of glioblastoma^9^,^10^. Salinosporamide A, a β-lactone-ɣ-lactam from *Salinispora tropica*, entered phase I clinical trials as a drug for treatment of multiple myeloma just three years after its discovery^11^.

The post-genomic era in actinomycete research is outlined by the discovery of multiple secondary metabolite biosynthesis gene clusters in the strains thought to be producing only few compounds^12^,^13^. This finding led to a rapid development of genomics-based approaches for the discovery of new natural products, resulting in isolation of new secondary metabolites from well-studied strains^14^,^15^. The application of genomics-based approaches has also broadened the diversity of natural products discovered from the marine actinomycetes^16^,^17^.

Here, we report the genome sequencing of the marine-derived actinomycete MP131-18. The detailed phylogenetic classification identified the strain as *Streptomyces*. The secondary metabolite biosynthesis gene clusters analysis and complete dereplication of secondary metabolites profile of the strain led to identification of several known and new natural products, their association with the corresponding gene clusters, and prediction of the biosynthetic pathways.

## Results

### General properties of the *Streptomyces* sp. MP131-18 genome

Strain MP131-18 was isolated from a deep-water marine sediment sample collected in the Trondheim fjord, Norway. The sediment suspension from which this isolate was obtained was treated with extremely high frequency radiation (EHF) that was shown to selectively promote growth of various rare actinomycete bacteria^18^. The MP131-18 isolate was recovered after plating of the diluted EHF-treated suspension on oatmeal agar. On this medium, the isolate had a cream-colored aerial mycelium, while its substrate mycelium and spores were brown with olive tinge. Its cell wall was shown to contain LL-diaminopimelic acid, which is characteristic for *Streptomyces* spp. Still, phenotypic characteristics of the isolate MP131-18 were not typical for streptomycetes, prompting phylogenetic analysis of its 16S rRNA gene. A 1421 bp PCR fragment obtained from the genomic DNA of MP131-18, representing the almost complete 16S rRNA gene, was sequenced and analysed using Ribosomal Database Project Classifier, which strongly suggested it belonging to the genus *Streptomyces*.

**Figure 1:**
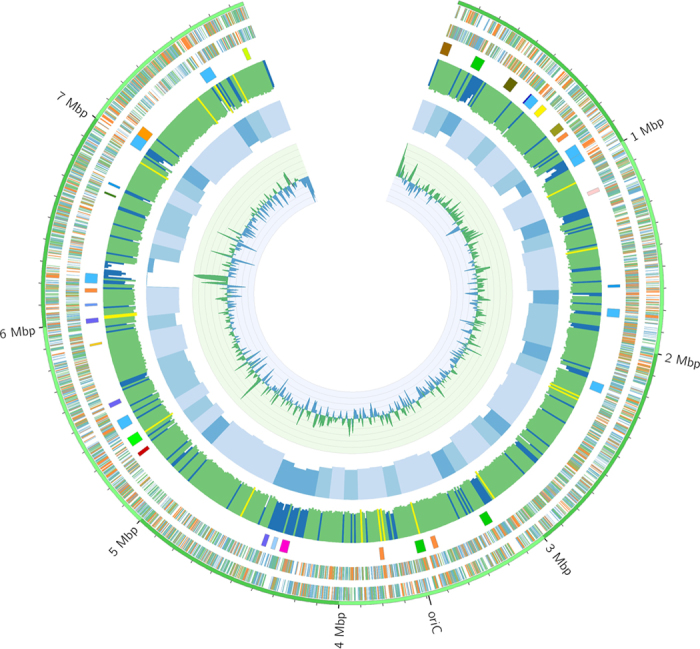
Schematic representation of the *Streptomyces*sp. MP131-18 genome (scaffold 1 only), created with the help of Circos^58^. Megabases are labeled; smaller ticks correspond to 100 kbp segments. From outside: genes on the forward and the reverse strands (blue: shorter than 900 bp, green: between 900 and 1500 bp long, orange: longer than 1500 bp); 35 secondary metabolite clusters colored by predicted type; G+C content, 10 kbp window (blue color highlights segments with G+C content <69%, yellow highlights correspond to G+C content over 76%); G+C content, 100 kbp window (lighter blue is higher G+C, darker blue is lower G+C content); G+C skew (green: positive; blue: negative).

**Figure 2:**
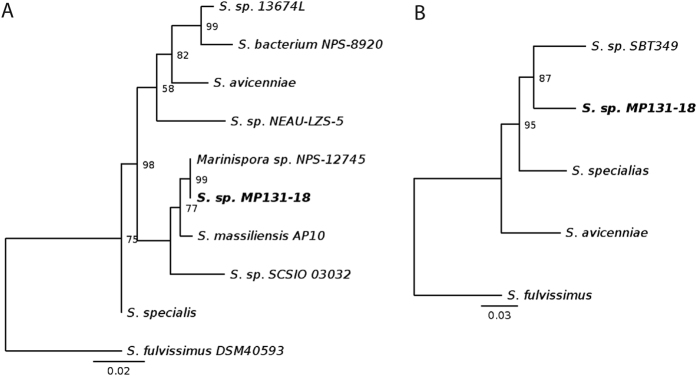
(**A**) Relation of *Streptomyces* sp. MP131-18 to the most 16S rRNA similar *Streptomyces. S. fulvissimus* 16S rRNA was used as outgroup. B. Relation of *Streptomyces* sp. MP131-18 to three other *Streptomyces*, as estimated by a 5-protein concatemer phylogenetic analysis. *S. fulvissimus* concatenated protein was used as outgroup.

Sequencing of the *Streptomyces* sp. MP131-18 genome was performed using two Illumina MiSEQ libraries – short-insert (paired-end, PE) and long-insert (mate pair, MP). The genome was assembled in a total of 10 scaffolds. The chromosome of *Streptomyces* sp. MP131-18 appears to be linear with most of the genome (7,861,428 bp) in scaffold 1 (Fig. 1). The other 9 scaffolds cover in total 95 kbp of genomic information with the largest being 48 kbp in size, and do not carry any housekeeping or secondary metabolism genes. Based on both PE and MP linking evidence (Figs 1S and 2S), the 9 extra scaffolds could represent unplaced parts of the scaffold 1 – that is, scaffold 1 is the complete bacterial chromosome, where gaps could be filled by the other 9 scaffolds. No plasmids were identified in *Streptomyces* sp. MP131-18. The G+C content (72.4%), the number of protein (7,054) and tRNA (78) encoding genes are comparable with other streptomycetes^12^. The genes for chromosomal replication initiation factor, *dnaA* (SBA_03553), and β-subunit of the DNA polymerase III, *dnaN* (SBA_03552), are located in the central part of the scaffold 1. The *oriC* is located between the *dnaN* and *dnaA* genes and contains three DnaA boxes (TTGTGCACAGG) conserved in *Streptomyces* species^19^. Both ends of scaffold 1 contain telomere-like ~100 bp inverted repeats with 4 possible hairpin structures.

The analysis of the genome sequence using the secondary metabolites biosynthesis genes search tool antiSMASH^20^ revealed the presence of 36 putative gene clusters (Fig. 1). In comparison, the genomes of *S. coelicolor*^12^, *S. averitilis*^13^, and *S. albus* J1074^21^ contain 20, 25 and 22 secondary metabolism gene clusters, respectively. The location of many of the secondary metabolism gene clusters in the *Streptomyces* sp. MP131-18 chromosome coincides with regions of low G+C content (Fig. 1).

### Phylogenetic analysis

To taxonomically delineate *Streptomyces* sp. MP131-18, we performed phylogenetic analysis by two complementary approaches using 16S ribosomal RNA sequences (Table 1S) and 5 genes coding for housekeeping proteins (RpoB, DnaK1, RecA, SsgB, and SsgA; Table 2S)^22^,^23^. A dendrogram based on the 16S rDNA gene (Fig. 2A) clearly shows that *Streptomyces* sp. MP131-18 is closely related to the unclassified actinobacterium NPS-12745 (GenBank accession EF551062.1), previously proposed to be a member of the new genus *Marinispora*^24^. There is only a single ambiguity in the aligned part of both 16S rRNAs: where *Streptomyces* sp. MP131-18 has a cytosine (1516 bp), NPS-12745 has an IUPAC ambiguity code for pyrimidine. The second closest hit that showed similarity to 16S rRNA from both *Streptomyces* sp. MP131-18 and *Streptomyces* sp. NPS-12745 is strain *Streptomyces massiliensis*, isolated from human gut^25^.

To perform an alternative protein-based phylogenetic analysis, the amino acid sequences of five highly conserved proteins (RpoB, DnaK1, RecA, SsgB, and SsgA; Table 2S)^22^,^23^ from MP131-18 were used for searches (via BlastP) against the NCBI NR database to identify the most similar proteins from other species. Three *Streptomyces* species were occurring the most often in search results for DnaK1, RecA, RpoB and SsgB: marine actinomycete *Streptomyces sp*. SBT349^26^, as well as the soil isolates *S. specialis*^27^, and *S. avicenniae*, both found in the rhizosphere of the mangrove plant^28^. SsgA search results had protein identities under 70% and thus were not considered. The five protein sequences from 4 genomes (MP131-18 and the three aforementioned species) and *S. fulvissimus* as an outgroup were concatenated, multiple-aligned using MAFFT, and used for RaxML dendrogram construction (Fig. 2B).

The phylogeny based on the five proteins suggests (and 16S rDNA based phylogeny supports) that *Streptomyces* sp. MP131-18 is most similar to *S. specialis* and *S. avicenniae*. Both protein- and 16S-based phylogenies identified representatives of the *Streptomyces* genus as neighbours of MP131-18. Thus, we conclude with a high degree of confidence that MP131-18 as well as NPS-12745 and the unclassified *Streptomycetaceae* bacterium NPS-8920 belong to the genus *Streptomyces*.

### Functional gene annotation

Functional annotation of *Streptomyces* sp. MP131-18 proteins with the bactNOG subset of the eggNOG v4 database (using protein BLAST with an expectation value cut-off of 0.001)^29^ resulted in identification of possible orthologues with some biological function assigned for 4,533 (64.3%) out of 7,054 proteins (Table 3S), with some of the genes assigned to more than one category. Of the remainder, 986 CDSs (14%) had no hits against bactNOGs, and 1,535 proteins (21.8%) had hits against proteins without functional category (*function unknown*). Among the proteins with functional assignment, 2,018 (28.6%) are implicated in metabolism, including 170 (2.4%) participating in secondary metabolism.

### Analysis of secondary metabolism gene clusters

The antiSMASH3.0 analysis of *Streptomyces* sp. MP131-18 genome revealed 36 gene clusters predicted to be involved in the secondary metabolism of the strain, occupying 8.4% of the chromosome. However, the re-evaluation of antiSMASH3.0 results revealed that cluster 1 is most probably encoding two distinct pathways for terpene and polyketide biosynthesis (designated as cluster 1a and 1b respectively) (Table 4S).

Within the genome of *Streptomyces* sp. MP131-18, six gene clusters with type I polyketide synthase (PKS) genes were predicted, most of them coding for small mono- or bimodular PKSs. Cluster 35 is probably involved in production of a compound containing an α-pyridone ring, based on high similarity to PKS and post-PKS enzymes from the piericidin A1 biosynthesis gene cluster^30^. Other type I PKS gene clusters (9, 10 and 33) have no homologues in sequenced bacterial genomes available in the public databases, and their products could be just partially predicted from the genes’ organization. Gene cluster 1b is coding for type II PKS most probably synthesizing compounds of the angucycline group. The presence of glycosyltransferase and sugar aminotransferase encoding genes in the 1b cluster suggests glycosylation of the produced polyketide. Gene cluster 7 is coding for the type III PKS that resembles the α-pyrone type PKS from fungi and plants^31^. A similar type III PKS is also found in the hybrid gene cluster 3, together with a monomodular type I PKS encoding gene. Cluster 19 is predicted to encode a biosynthetic pathway for a polyunsaturated aryl polyene-like compound. Only three non-ribosomal peptide synthase (NRPS) gene clusters were found within the genome of *Streptomyces* sp. MP131-18 (2, 15, 17). Cluster 2 is similar to the coelibactin gene cluster from *S. coelicolor*^12^. The structure of this compound is not known, but it is thought to act as zincophore. Four gene clusters - 13, 14, 30, and 34 - combine type I PKS and NRPS-encoding genes. Clusters 13, 14, and 30 include genes coding for discrete adenylation domain proteins that most probably are supplying the amino acid starter units for the PKS components.

Five gene clusters (1a, 8, 16, 18, 29) within the genome of *Streptomyces* sp. MP131-18 are devoted to terpenes biosynthesis, including cluster 18 for the sesquiterpene geosmin. Cluster 23 is coding for a type I PKS and a terpene synthase/cyclase. It also contains a glycosyltransferase suggesting that the putative hybrid product might be glycosylated.

Another large group of secondary metabolism gene clusters are coding for ribosomally synthesized and post-translationally modified peptides (RiPPs)^32^. Clusters 6, 21, 25, and 27 are coding for lantipeptide type compounds. Cluster 6, beside the *lanM* homologue, also harbours a gene for thiazole ring formation. Cluster 20 is similar to a recently discovered actinobacterial gene cluster for lasso-peptide biosynthesis^33^.

Several other secondary metabolites could be potentially produced by *Streptomyces* sp. MP131-18. Among them, two siderophore molecules encoded by clusters 12 and 32 (cluster 32 showed high similarity to desferrioxamine B biosynthesis genes), melanine (cluster 26), ectoine (cluster 31), bacteriocin (cluster 28), and A-factor like butyrolactone (cluster 4). Cluster 11 is presumably governing biosynthesis of a pyrrolopyrimidine nucleoside antibiotic. Two other gene clusters, 22 and 36, are predicted to be responsible for phenazine and indolocarbazole type compounds biosynthesis, respectively.

### *Streptomyces* sp. MP131-18 produces a group of bisindole pyrrole antibiotics

Based on the genome analysis, *Streptomyces* sp. MP131-18 appeared to be a strain with prolific potential for production of various, potentially new, secondary metabolites. In order to assess this, MP131-18 was grown in two different production media, and extracted metabolites were found to be active against *Bacillus subtilis* but not *Pseudomonas putida*. The extracts were further analysed by high-resolution LC-QTOF mass spectrometry and compounds were dereplicated using the Dictionary of Natural Products (DNP) database^34^.

**Figure 3:**
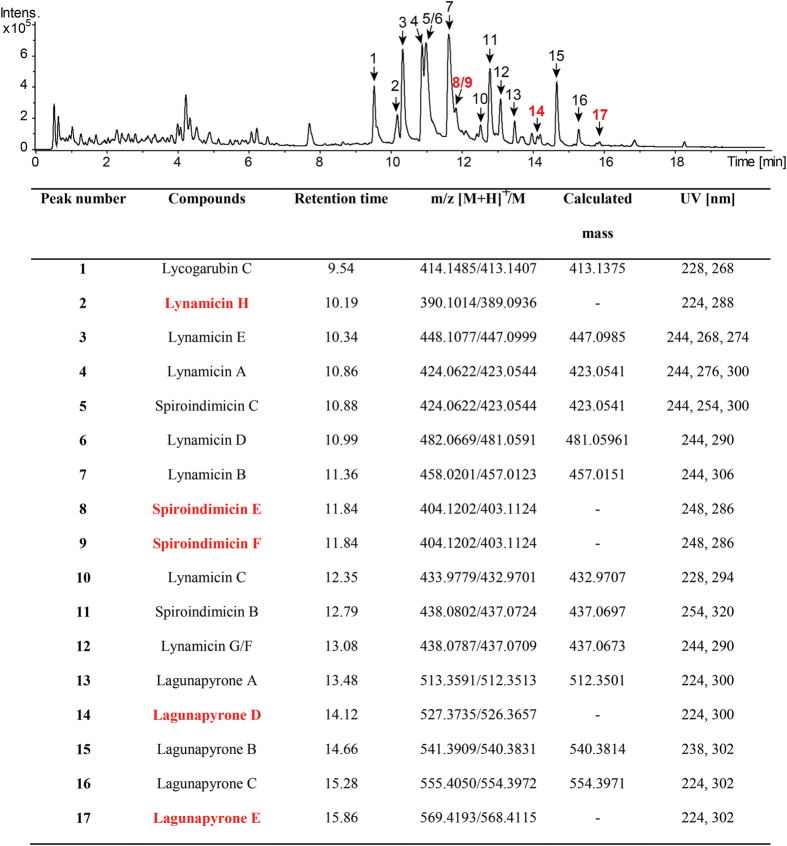
DAD chromatogram of extracts from *Streptomyces* sp. MP131-18. The identified compounds are indicated by numbers and their features are listed in the table. Compounds identified during this work are highlighted in red.

**Figure 4:**
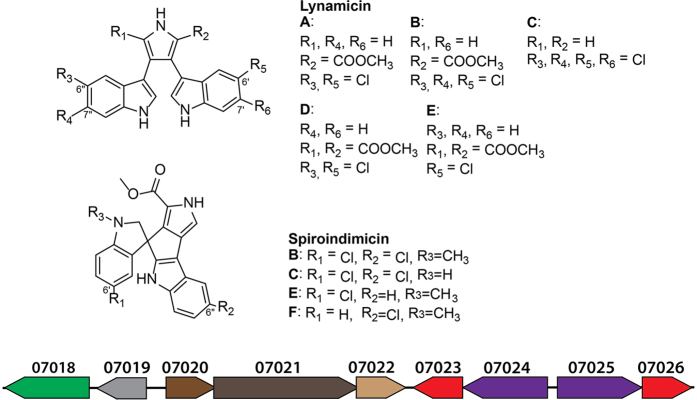
Structures of bisindole pyrrole compounds identified in the extract from *Streptomyces* sp. MP131-18 and schematic representation of gene cluster 36. Predicted genes functions: SBA_07018 – transcriptional regulator; SBA_07019 – putative transporter protein; SBA_07020 - tryptophan 2-monooxygenase; SBA_07021 – chromopyrrolic acid synthase, VioB homologue; SBA_07022 – RebP-like cytochrome P450; SBA_07023, SBA_07026 - flavin reductases; SBA_07024, SBA_07025 – halogenases.

The dominant metabolites produced by *Streptomyces* sp. MP131-18 were two groups of closely related bisindole pyrrole antibiotics, lynamicins and spiroindimicins (Fig. 3). Based on the exact masses and absorption spectra, we were able to identify lynamicins A-G (peaks 2-4, 6, 7, 10, 12) and spiroindimicins B and C (peaks 11, 5) (Figs 3 and 4). These compounds were previously isolated from the marine actinomycete *Streptomyces* sp. SCSIO 03032 and a strain designated as *Marinispora* NPS-12745^24^,^35^,^36^. Both lynamicins and spiroindimicins were shown to have antibacterial activity. Thus, the ability of the *Streptomyces* sp. MP131-18 extracts to inhibit growth of *B. subtilis* is most probably caused by accumulation of these compounds. Lynamicin E and spiroindimicin B were purified (4.8 mg and 2.9 mg from 5L of culture, respectively) and their structures confirmed by NMR. 1D ^1^H, ^13^C NMR spectra and 2D correlations of ^1^H-^1^H COSY, HSQC and HMBC NMR spectra are summarized in Supplementary Tables 5S and 6S. Moreover, the compound **1** was identified as lycogarubin C^37^, also known as chromopyrrolic acid, the key intermediate in bisindole pyrroles biosynthesis (Figs 3 and 3S)^38^.

The compound with RT of 11.84 min showed the absorption spectra typical for identified bisindole pyrroles, but the detected mass did not correspond to any known lynamicins or spiroindimicins (Fig. 3)^24^,^35^,^36^. During the HPLC, the peak was found to separate into two peaks (Fig. 4S), and both of these compounds **8** and **9** were purified and their structures elucidated by NMR.

The compounds **8** and **9** were obtained as white solids (yield: 0.3 mg and 0.6 mg from 5 L of culture, respectively). The HRESIMS of compound **9** gave a quasimolecular ion peak at *m/z* 404.1202 [M+H]^+^ corresponding to a molecular formula of C_23_H_18_ClN_3_O_2_ (Fig. 3). The UV spectrum of **9** with maxima at 248 and 286 nm was similar to other spiroindimicins. Compound **9** displayed the same molecular ion peak at HRESIMS (*m/z* 404.1202 [M+H^+^]), UV absorption spectra (λ_max_ 248 and 286 nm) and molecular formula C_23_H_18_ClN_3_O_2_ as **8,** but had slightly different retention time. The new compounds were named spiroindimicin E (**8**) and spiroindimicin F (**9**). The 35Da mass difference of both new compounds to spiroindimicin B let us assume that the disparity is caused by the lack of one chlorine atom. This was confirmed by the analysis of the isotopic pattern of mass peaks of spiroindimicins B, E and F (data not shown). Furthermore the same *m/z* at HRESIMS and UV spectra for the new compounds suggest that they are constitutional isomers (Figs 3 and 4).

2D NMR data of both spiroindimicins E and F showed high similarity to those of spiroindimicin B (Tables 7S and 8S). Careful analysis of protons and carbons in the aromatic region allowed us to assign the position of chlorine atoms. In fact, the ^1^H NMR spectrum of compound **8** exhibited eight aromatic signals. Proton splitting pattern of four of them and their HMBC correlations suggested compound **8** lacks a chlorine atom at C-6” in comparison to spiroindimicin B (Table 6S). Furthermore, the remaining two meta coupled doublets revealed the chlorine atom is at C-6’ in spiroindimicin E (Table 7S). This finding was further supported by the HMBC correlations. Sequential COSY correlations of protons H5’-H8’ and HMBC correlations of spiroindimicin F suggested the indol ring comprising protons H5’-H8’ is not substituted, while the remaining indol ring has chlorine substitution at C6” due to proton splitting patterns as well as HMBC correlations (Table 8S).

Another minor compound with RT of 10.19 min was obtained in scarce amounts and it exhibited a quasimolecular ion peak at m/z 390.1014 [M+H]^+^. This compound is predicted to be monochlorinated lynamicin F-like bisindole pyrrole based on its HRESIMS and isotopic pattern (Fig. 3). However, further investigation is required to establish the exact structure of this metabolite, tentatively designated as lynamicin H.

Isolated compounds (lycogarubin C, lynamicin E, spiroindimicin B and spiroindimicin E) were tested against *Bacillus subtilis, Escherichia coli* and *Pseudomonas putida* (Table 9S). None of them were found to be active against Gram negative test cultures and only spiroindimicin B demonstrated moderate activity against *Bacillus subtilis.* The antibacterial properties of spiroindimicins, similar to lynamicins^24^, seem to correlate with the degree of chlorination. The monochlorinated lynamicins and spiroindimicins are weak inhibitors of bacterial growth, while antibacterial activity of both families of compounds is increasing with the increase of number of halogen atoms in their structures.

**Figure 5:**
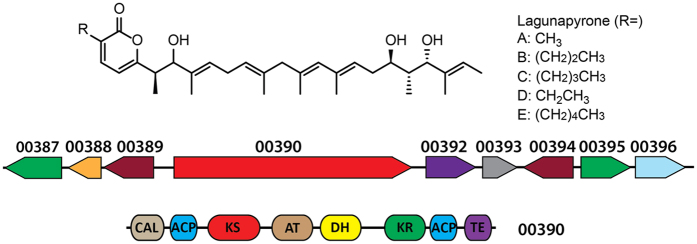
Structure of lagunapyrones A-E and schematic representation of gene cluster 3. The domain organization of putative lagunapyrone iPKS (SBA_00390) is predicted by antiSMASH 3.0^20^.

One of the new compounds, spiroindimicin E, as well as previously described lynamicin E and lycogarubin C were tested for cytotoxic activity (Fig. 5S). Spiroindimicin E shows a very slight effect on cellular growth of T24 bladder carcinoma cells, whereas treatment with 10 μM lynamicin E led to more than 50% reduction of proliferation capacity. At the same time, as expected, lycogarubin C almost completely inhibited proliferation of the tested cancer cell line at this concentration.

Among the secondary metabolite biosynthesis gene clusters, the only candidate identified for bisindole pyrroles biosynthesis is cluster 36, thereafter called *lyn* (Fig. 4, Table 4S). The core genes of the cluster, designated SBA_07020, SBA_07021 and SBA_07022, are coding for tryptophan 2-monooxygenase, chromopyrrolic acid synthase, and cytochrome P450, respectively. These genes are known to be involved in assembly of bisindole pyrrole core and probably its closing into spiroindimicins structures. Genes SBA_07024 and SBA_07025 are encoding two halogenases that presumably attach chlorine atoms at two different positions on the bisindole rings (6’/6” and 7’/7”) (Fig. 4). The halogenase genes are forming operons with the genes SBA_07022 and SBA_07026 coding for flavin reductases participating in halogenation reaction. Lastly, SBA_07018 is encoding a transcriptional regulator of the NarP family, probably controlling the expression of the structural genes, and SBA_07019 is encoding a putative transporter protein probably involved in export of metabolites from the cell.

### *Streptomyces* sp. MP131-18 produces unusual α-pyrones

**Figure 6:**
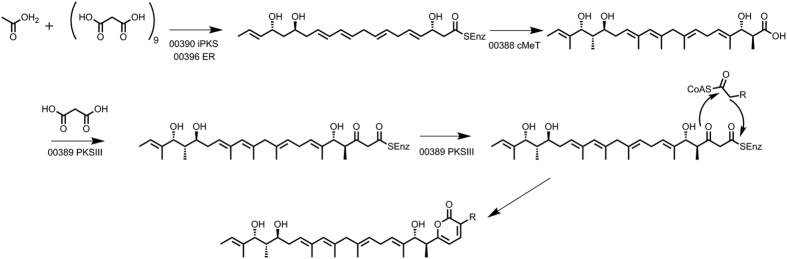
Proposed scheme for lagunapyrones A-D biosynthesis.

Beside the bisindole pyrroles, *Streptomyces* sp. MP131-18 was found to accumulate other compounds belonging to the α-pyrone group. Three lagunapyrones A-C were identified by exact mass and absorption spectra in the extracts from *Streptomyces* sp. MP131-18 culture (Figs 3 and 5). These compounds were originally isolated from the unclassified marine actinomycete CNB-984, and comprise a group of closely related α-pyrones functionalized by a highly methyl-branched C19 side chain^39^. Despite the structural similarity of α-pyrones, they can be assembled by PKS enzymes of either type I, II or III. The best candidate for lagunapyrones biosynthesis within the genome of *Streptomyces* sp. MP131-18 is cluster 3, which contains genes coding for a single module type I PKS (SBA_00390) and α-pyrone type III PKS enzymes (SBA_00389). The possible implication of the type III PKS allowed us to predict the high flexibility of the choice of acyl-CoA esters that are utilized by the pathway. Based on this prediction, we were able to identify two new lagunapyrones D and E produced by *Streptomyces* sp. MP131-18. The newly identified metabolites have MS2 fragmentation patterns similar to the previously identified compounds A-C (Fig. 6S). The characteristic daughter ion peak at *m/z* 321.3 [M+H]^+^ was observed for all lagunapyrones, which is ascribable to the side chain cleaved between C6 and C7 after loss of three water molecules. Lagunapyrones D and E differ from the described A and C congeners, respectively, by 14Da, most probably indicating a differences in the alkyl chain length at C2 position of α-pyrone ring (Fig. 5).

## Discussion

The discovery of new antibiotics faced the stagnation phase when scientists realized that the same compounds are more and more often isolated from different actinomycete bacteria obtained from soil. Realizing this phenomenon caused a rapid change in the strategies used for natural product discovery by switching to new sources in the search of producing organisms. Undoubtedly, the oceans are the largest source of biodiversity and are relatively poorly studied. The growing information on the biosynthetic potential of marine microorganisms further invigorated the research in this area^6^,^8^.

Here we report the use of a combined genomics-metabolomics approach in order to identify and isolate new compounds from a marine-derived actinomycete. Strain MP131-18 was isolated from a deep-water sediment sample and its genome was sequenced. The genome size, structure, G+C content and other features are typical for other streptomycetes genomes (Fig. 1). Both 16S rRNA and multilocus marker phylogenetic analyses clearly showed that the strain belongs to the genus *Streptomyces* (Fig. 2). The strain was found to be closely related to another marine actinomycete previously classified as *Marinispora* sp. NPS-12745^24^. Interestingly, several regions in the *Streptomyces* sp. MP131-18 genome have lower than average G+C content (Fig. 1). These regions comprise putative genomic islands, the recently restructured parts of the chromosome, most likely acquired by horizontal gene transfer^40^. The location of the secondary metabolism gene clusters within these putative genomic islands suggests that their origin is most likely other microorganisms, including *Proteobacteria* and fungi.

For a streptomycete, *Streptomyces* sp. MP131-18 possesses an average number of gene clusters dedicated to secondary metabolism: 36 clusters, occupying 8.4% of the genome. This number is slightly higher than in *S. coelicolor* A3(2) (20 gene clusters)^12^ or *S. avermitilis* MA-4680 (25 gene clusters)^13^ with 5% and 6.6% of the genes governing secondary metabolism biosynthesis, respectively, but still lower than in the case of *Kutzneria albida* with 14% of the chromosome devoted to secondary metabolism^41^. The *Streptomyces* sp. MP131-18 genome is encoding clusters for the synthesis of at least 18 different types of secondary metabolites, proving the high potential of marine actinomycetes to produce chemically diverse compounds. These include genes for a lassopeptide that is just a second example among actinobacteria^33^ and aryl polyenes that were found so far only in *Proteobacteria*^42^. The latter compounds are thought to act as photoprotecting and antioxidant agents.

The gene cluster 36, due to similarity to rebeccamycin biosynthesis genes, was predicted to be involved in the biosynthesis of an indolocarbazole type compound. Indeed, two groups of highly halogenated bisindole pyrroles, lynamicins and spiroindimicins, were identified in the extracts of *Streptomyces* sp. MP131-18 (Fig. 3). Beside lynamicins A-G and spiroindimicins A-D, previously isolated from the marine actinomycetes *Streptomyces* sp. SCSIO 03032 and *Marinispora* NPS-12745^24^,^35^,^36^, we have purified two new compounds designated spiroindimicin E and F (Figs 3 and 4). Tentatively, a new lynamicin H was also identified, but was not structurally characterized. Besides that, lycogarubin C can be found in the *Streptomyces* sp. MP131-18 extract^37^. This metabolite was originally discovered in the extract of the slime mould *Lacogala epidendrum*, and later as an intermediate in staurosporine and rebeccamycin biosynthesis in *S. longisporoflavus* and *Lechevalieria aerocolonigenes* A, respectively^43^,^44^. The presence of lycogarubin C in the extracts of *Streptomyces* sp. MP131-18 and its structural resemblance to the core of the identified antibiotics suggest that it might act as a common precursor or a shunt product in the biosynthesis of lynamicins and spiroindimicins. The fact that halogenation occurs before the assembly of the bisindole pyrrole ring system supports the shunt product hypothesis (Figs 3 and 7S)^45^. This indicates the high flexibility in substrate selection by the bisindole pyrrole assembly enzymes. Because of the structural diversity of lynamicins and spiroindimicins, the identified gene cluster might comprise a source of individual genes for the combinatorial biosynthesis of new bisindole pyrrole and indolocarbazole antibiotics, especially the ones encoding halogenation steps and formation of spirane structure.

A group of α-pyrones called lagunapyrones were also identified in the extract of MP131-18 (Fig. 5)^39^. These compounds comprise a long methyl-branched polyketide chain assembled into the final molecule by the linkage to the short fatty acid chain through the pyrone ring. The only gene cluster that could be involved in the biosynthesis of such compounds (cluster 3) contains type I and type III PKS genes. The type I PKS resembles the iterative highly reductive PKS enzymes from the fungal plant pathogen *Alternaria solani*, involved in alternapyrone biosynthesis, and is thought to synthesise the C20 polyketide chain (Fig. 6)^46^. Furthermore, the predicted lagunapyrone iPKS does not contain the enoylreductase domain. This activity is thought to be supplied by separate enzymes similar to those involved in the biosynthesis of the fungal metabolite lovastatin^47^. The acyl-transferase domains of PKSI enzymes are predicted to be specific for malonate extender units. The highly methyl-branched C19 polyketide side chain of lagunapyrones is assumed to be decorated as a post-PKS modification similar of the corresponding steps in alternapyrone or nafuredin biosynthesis^46^,^48^. However, the lagunapyrone PKSI is lacking the C-methyltransferase domain, while a gene coding for standalone C-methyltransferases is located in close proximity to the PKSIII gene. The type III PKS is presumably further extending the C20 polyketide chain assembled by PKSI with a single acetate unit. The same enzyme is thought to perform the intermolecular closing of α-pyrone ring with the short chain fatty acid-CoA esters^49^. Taking into account the flexibility of type III PKS enzyme in choosing the acyl-CoAs, we predicted the existence of at least two other lagunapyrones with C2 and C5 alkyl chains. Indeed, the corresponding compounds, named as lagunapyrone B and E, were identified in the extract of *Streptomyces* sp. MP131-18 (Figs 3 and 5). This finding proves that the combination of detailed metabolomics and genomics is a powerful tool in finding new natural products. The prediction and understanding of biosynthetic processes leading to formation of particular metabolites helps to identify these compounds.

## Methods

### Bacterial strains, cultures conditions and general procedures

*Streptomyces* sp. MP131-18 was isolated from a marine sediment collected at the depth of 450 m at the Tautra ridge in the Trondheim fjord, Norway (63°55,909N, 010°61,846 E). The sediment sample was treated with extremely high frequency radiation and plated in dilutions on the oatmeal agar as described in^18^. *Streptomyces* strains were grown on mannitol soy flour agar (MS agar) and in liquid TSB medium^50^. NL19 (MS medium without agar)^50^ and SG^51^ mediums were used for secondary metabolite production.

### Genome sequencing

For DNA isolation, *Streptomyces* sp. MP131-18 strain was inoculated into TSB medium and grown at 28 °C with shaking (200 rpm) for 3 days. High quality total cellular DNA was isolated using salting out procedure^50^. The purity and concentration of the genomic DNA was determined using a Nanodrop 2000 spectrophotometer (Thermo Fisher Scientific).

The obtained genomic DNA was sequenced using two MiSEQ libraries (Illumina) – short-insert (paired-end) and long-insert (mate pair). Assembly of the shotgun reads was performed with the Newbler v2.8 assembler (Roche). Genome annotation was performed using Prokka^52^. All predicted ORFs were manually re-inspected to correct start codon position. For the identification of secondary metabolites clusters antiSMASH 3.0 was used^20^.

### Nucleotide sequence accession number

The *Streptomyces* sp. MP131-18 Whole Genome Shotgun project has been deposited at DDBJ/ENA/GenBank under the accession LZNS00000000. The version described in this paper is version LZNS01000000.

### Phylogenetic analysis

rRNA delineation was initially performed using the ARB-SILVA database^53^, which suggested a number of neighbour (most similar) species. 16S rRNA sequences of these species were downloaded from ARB-SILVA (Table 1S), and multiple-aligned using MAFFT v7.222^54^ (algorithm: auto, scoring matrix: 200PAM/k = 2, gap open penalty 1.53, offset value 0.123). The dendrogram was constructed using RaxML 7.2.8^55^ (nucleotide model: GTR GAMMA, algorithm: rapid bootstrapping and search for best-scoring ML tree, bootstrap replicates: 1000). The tree was formatted in Geneious 9.0.4^56^.

MAFFT and RAxML were also used for 5-gene alignments, with the same options as above. Accession numbers of the used gene sequences are listed in Table 2S.

### Strain cultivation and metabolites extraction

The pre-cultures were grown in 10 ml of TSB in 100 mL flasks with glass beads for 3 days at 28 °C. Subsequently, the main cultures (50 ml of SG or NL19 medium in 500 ml flasks) were inoculated with 4 ml of pre-culture and cultivated at 28 °C on a rotary shaker at 180 rpm for 7 days. Metabolites were extracted with acetone:methanol 1:1 from biomass and with ethyl acetate from cultural liquid, evaporated and dissolved in 250 μl of methanol. The LC-HRMS data were collected on a Dionex Ultimate 3000 RSLC system using a BEH C18, 100 × 2.1 mm, 1.7 μm d_p_ column (Waters, Germany). Separation of 1 μl sample was achieved by a linear gradient of solvent B (acetonitrile with 0.1% of formic acid) against solvent A (water with 0.1% of formic acid) at a flow rate of 600 μl/min and 45 °C. The gradient started by a 0.5 min isocratic step at 5% B then increased to 95% B over 18 min to end up with a 2 min step at 95% B before re-equilibration under the initial conditions. UV spectra were acquired by a DAD in the range of 200 to 600 nm. The mass-spec data was collected on a maXis 4 G hr-ToF ultrahigh resolution mass spectrometer (Bruker Daltonics, Germany) using the Apollo II ESI source. Mass spectra were acquired in centroid mode ranging from 200 to 2500 m/z at a 2 Hz scan rate. Extracts from biomass and supernatant were analysed separately.

Data were collected and analysed with the Bruker Compass Data Analysis software, version 4.1 (Bruker, Billerica, USA). The screening for known compounds was performed using the Dictionary of Natural Products database version 6.1 (CRC Press, Boca Raton, USA), using the following parameters: accurate molecular mass (mass match tolerance 10 ppm), absorption spectra and source of compounds isolation^34^.

### Isolation and purification of compounds

*Streptomyces* sp. MP131-18 was grown at 28 °C for 3 days in 6 × 500 ml flasks containing 50 ml of TSB medium and pre-cultures were used to inoculate 100 × 500 ml flasks containing 50 ml of SG media. Cultures were incubated at 28 °C for 7 days on rotary shakers at 180 rpm. The biomass and cultural liquid were separated by centrifugation. Metabolites were extracted from supernatant with 3L of ethyl acetate and from biomass with 500 ml of a 1:1 mixture of acetone:methanol. Extracts were evaporated and resuspended in 20 ml of methanol. Extracts from biomass and supernatant were combined.

Extracts were fractionated in two steps: size-exclusion chromatography and preparative HPLC. The extracts were loaded on a 1 m long column packed with LH 20 Sephadex (Sigma-Aldrich) and eluted with methanol as solvent. The fractions were collected every 15 min with a flowrate of 1 ml per minute. The obtained fractions were evaporated and dissolved in 0.5 ml of MeOH.

Samples were further separated by preparative HPLC (Agilent 1100 Series, Agilent Technologies and Dionex UltiMate 3000, Thermoscientific) using NUCLEODUR^®^ C18 HTec column (250 × 10 mm, 5 μm) (Macherey-Nagel) with a linear gradient of solvent B (acetonitrile with 0.1% of formic acid) against solvent A (water with 0.1% of formic acid) at a flow rate of 4.5 ml/min and 45 °C. New compounds were separated using a gradient starting from 30% and increasing to 70% of B over 30 min. UV spectra were recorded with DAD detector at 280 nm. Individual peaks were collected and analysed with a LC-MS amaZon system (Bruker, Daltonics) using a BEH C18, 50 × 2.1 mm, 1.7 μm d_p_ column (Waters, Germany) and a linear gradient 5-95% of B over 9 min.

NMR spectra were acquired on a Bruker Ascend 700 MHz NMR spectrometer equipped with a 5 mm TXI cryoprobe. As solvent, deuterated MeOD_4_ and DMSO-d_6_ were used and HSQC, HMBC, ^1^H-^1^H COSY spectra were recorded using standard pulse programs.

### Antibacterial assay

Antimicrobial activities of the extracted metabolites were assayed by a disc diffusion method, loading 40 μl of each extract on 6 mm diameter paper discs. Test cultures of *Bacillus subtilis* ATCC 6633 and *Pseudomonas putida* KT 2440 were plated from the liquid cultures on solid LB medium, dried for 20 minutes prior applying the discs. Plates were incubated overnight at 37 °C. The zones of inhibition were measured manually with accuracy ±1 mm. The minimum inhibitory concentration (MIC) determination was conducted as described^57^, except that LB medium was used to grow the test cultures. Solution of thiazolyl blue tetrazolium bromide (Sigma-Aldrich) was used for growth visualization.

### Cell proliferation assays

T24 bladder carcinoma cells were stimulated for 72 h and stained with crystal violet solution (0.5% crystal violet (w/v), 20% methanol (v/v) in H_2_O). Next, unbound dye was removed by rinsing with distilled water. Following air-drying, crystal violet was dissolved in ethanol/sodium-citrate solution (50% ethanol (v/v), 0.1M sodium-citrate) and the absorbance was measured at 540 nm at day 0 to obtain the initial amount of living cells and after 72 h in order to calculate the proliferation rate. Data are expressed as mean ± SEM and analyzed using two-way ANOVA followed by Bonferroni’s multiple comparisons test. *p < 0.05, **p < 0.001, ***p < 0.001.

## Additional Information

**How to cite this article**: Paulus, C. *et al*. New natural products identified by combined genomics-metabolomics profiling of marine *Streptomyces* sp. MP131-18. *Sci. Rep.* **7**, 42382; doi: 10.1038/srep42382 (2017).

**Publisher's note:** Springer Nature remains neutral with regard to jurisdictional claims in published maps and institutional affiliations.

## Supplementary Material

Supplementary Materials
