# Supplementary material for: New natural products identified by combined genomics-metabolomics profiling of marine Streptomyces sp. MP131-18
Source: Sci Rep. 2017 Feb 10;7:42382. doi: 10.1038/srep42382 (PMC5301196; doi:10.1038/srep42382)
Supplement: Supplementary Materials [file srep42382-s1.doc]

**Supplementary information.**

**New natural products identified by combined genomics-metabolomics profiling of marine *Streptomyces* sp. MP131-18.**

Constanze Paulus^1^, Yuriy Rebets^1^, Bogdan Tokovenko^1^, Suvd Nadmid^1^, Larisa P. Terekhova^2^, Maksym Myronovskyi^1^, Sergey B. Zotchev^3,4^, Christian Rückert^5#^, Simone Braig^6^, Stefan Zahler^6^, Jörn Kalinowski^5^, Andriy Luzhetskyy^1,7^*

^1^Helmholtz-Institute for Pharmaceutical Research Saarland, Actinobacteria Metabolic Engineering Group, Saarbrücken, Germany

^2^Gause Institute of New Antibiotics, Russian Academy of Medical Sciences, Moscow, Russia

^3^Department of Biotechnology, Norwegian University of Science and Technology, Trondheim, Norway

^4^Department of Pharmacognosy, University of Vienna, Vienna, Austria

^5^Center for Biotechnology, Bielefeld University, Bielefeld, Germany

^6^Department of Pharmacy - Center for Drug Research, University of Munich, Munich, Germany

^7^Universität des Saarlandes, [Pharmaceutical Biotechnology](http://www.uni-saarland.de/en/campus/faculties/professorial-staff/naturwissenschaftlich-technische-fakultaet-iii/pharmazie/professorial-staff-dept-82-pharmacy.html), Saarbrücken, Germany

#present address: Department of Biology, Massachusetts Institute of Technology, Cambridge, MA, USA

*Corresponding author:

Prof. Dr. Andriy Luzhetskyy

E-mail: a.luzhetskyy@mx.uni-saarland.de

Universität des Saarlandes, [Pharmaceutical Biotechnology](http://www.uni-saarland.de/en/campus/faculties/professorial-staff/naturwissenschaftlich-technische-fakultaet-iii/pharmazie/professorial-staff-dept-82-pharmacy.html), Building C2.3, 66123 Saarbrücken, Germany.

**Table 1S.** 16S ribosomal DNA sequences used for 16S phylogenie.

| Species | ARB-SILVA accession / rRNA locus_tag | Identity with the SBA_07180, % |
| --- | --- | --- |
| *Streptomyces* sp. MP131-18 | SBA_07180 | - |
| *Streptomyces fulvissimus* DSM40593 | SFUL_2616 | 92.53 |
| *Streptomyces* sp. 13674L | EU741227.1.1494 | 96.67 |
| *Streptomycetaceae bacterium* NPS-8920 | EF470589.1.1523 | 96.43 |
| *Streptomyces avicenniae* | EU399234.1.1491 | 96.4 |
| *Streptomyces* sp. NEAU-LZS-5 | KC304791.1.1538 | 96.09 |
| *Marinispora* sp. NPS-12745 | EF551062.1.1533 | 99.97 |
| *Streptomyces massiliensis* AP10 | JX101691.1.1502 | 99 |
| *Streptomyces specialis* | AM934703.1.1500 | 97.35 |
| *Streptomyces.* sp. *SCSIO 03032* | JN798514.1 | 93.88 |

For *S. fulvissimus* and *S.* sp. MP131-18, locus_tags are provided; for *Streptomyces.* sp. *SCSIO 03032* GenBank accession number; other IDs are from ARB-SILVA database.

**Table 2S**. Table of compared gene sequences (locus_tags).

| Protein | *S.* sp. MP131-18 | *S. fulvissimus* | *S. specialis* | *S.* sp. SBT349 | *S. avicenniae* |
| --- | --- | --- | --- | --- | --- |
| RpoB | SBA_02824 | SFUL_RS21975 | BN2279_RS23020 | ADY01_RS15195 | IF27_RS10465 |
| DnaK1 | SBA_03307 | SFUL_RS16290 | BN2279_RS14960 | ADY01_RS04030 | IF27_RS03445 |
| RecA | SBA_01513 | SFUL_RS28035 | BN2279_RS19870 | ADY01_RS00075 | IF27_RS21960 |
| SsgB | SBA_03814 | SFUL_RS15590 | BN2279_RS17305 | ADY01_RS23410 | IF27_RS03120 |
| SsgA | SBA_04743 | SFUL_RS12465 | BN2279_RS22425 | ADY01_RS28310 | IF27_RS06350 |

**Table 3S**. Functional annotation of *Streptomyces* sp. MP131-18 genes.

| INFORMATION STORAGE AND PROCESSING | **1068** |
| --- | --- |
| Translation, ribosomal structure and biogenesis | 170 |
| RNA processing and modification  Transcription  Replication, recombination and repair  Chromatin structure and dynamics | 1  677  219  1 |
| CELLULAR PROCESSES AND SIGNALING | **846** |
| Cell cycle control, cell division, chromosome partitioning  Defense mechanisms  Signal transduction mechanisms  Cell wall/membrane/envelope biogenesis  Intracellular trafficking, secretion, and vesicular transport  Posttranslational modification, protein turnover, chaperones | 38  141  303  204  28  132 |
| METABOLISM | **2,018** |
| Energy production and conversion  Carbohydrate transport and metabolism  Amino acid transport and metabolism  Nucleotide transport and metabolism  Coenzyme transport and metabolism  Lipid transport and metabolism  Inorganic ion transport and metabolism  Secondary metabolites biosynthesis, transport and catabolism | 315  483  355  95  151  162  287  170 |
| POORLY CHARACTERIZED | **2,227** |
| General function prediction only  Function unknown | 692  1,535 |

**Table 4S.** Secondary metabolism gene clusters classified by the type.

| **Cluster type** | **Number of clusters** | **Clusters ID** |
| --- | --- | --- |
| PKSI | 6 | 5, 9, 10, 24, 33, 35 |
| PKSII | 1 | 1b |
| PKSIII | 1 | 7 |
| PKSI-PKSIII | 1 | 3 |
| NRPS | 3 | 2, 15, 17 |
| PKS-NRPS | 4 | 13, 14, 30, 34 |
| Terpene | 5 | 1a, 8, 16, 18, 29 |
| PKSI-Terpene | 1 | 23 |
| RiPPs | 5 | 6, 20, 21, 25, 27 |
| Nuceloside | 1 | 11 |
| Butyrolactone | 1 | 4 |
| Siderophore | 2 | 12, 32 |
| Arylpolyene (PKS) | 1 | 19 |
| Phenazine | 1 | 22 |
| Melanin | 1 | 26 |
| Bacteriocin | 1 | 28 |
| Ectoine | 1 | 31 |
| Indole | 1 | 36 |

**Table 5S.** NMR data of Lynamicin E.

| **position** | **δ_H_ (J in Hz)** | **δ_C_** | **Cosy** | **HMBC** |
| --- | --- | --- | --- | --- |
| **1** | NH | - | - | - |
| **2** | - | 122.9 | - | - |
| **3** |  |  |  |  |
| **4** |  |  |  |  |
| **5** | - | 122.8 | - | - |
| **6** | - | 161.3 | - | - |
| **7** | 3.69^a^ s | 50.6 |  |  |
| **8** | 3.66^a^ s | 50.4 | - | - |
| **9** | - | 161.7 | - | - |
| **1’** | NH | - | - | - |
| **2’** | 6.90 s | 126.3 | - | 3’ |
| **3’** | - | 108.0 | - | - |
| **4’** | - | 128.8 |  | - |
| **5’** | 7.02 d (1.7) | 118.6 | - | 9’-6’-7’ |
| **6’** | - | 124.4 | - | - |
| **7’** | 6.92 dd (2.0, 8.6) | 120.5 | 7.18 | 5’ |
| **8’** | 7.18 dd (0.4, 8.6) | 111.7 | 6.92 | 4’-6’ |
| **9’** | - | 134.2 | - | - |
| **1’’** | NH | - | - | - |
| **2’’** | 6.87 s | 124.4 | - | 3‘‘-4‘‘-9‘‘ |
| **3’’** | - | 107.9 | - | - |
| **4’’** | - | 127.7 | - | - |
| **5’’** | 7.07 d (7.9) | 119.5 | 6.80 | 3‘‘-9‘‘ |
| **6’’** | 6.80 t (0.7, 7.6) | 118.3 | 6.97-7.07-7.25 | 8‘‘-4‘‘ |
| **7’’** | 6.97 ddd (1.0, 7.7) | 118.9 | 6.80-7.25 | 5’’-9’’ |
| **8’’** | 7.25 d (8.1) | 110.6 | 6.97 | 6’’-4’’ |
| **9’’** | - | 136.1 | - | - |

Sample was measured in MeOH. Data were recorded on Bruker Ascend 700 MHz. Spectra referenced to internal solvent for MeOH at 3.31 ppm (^1^H) and 49.0 ppm (^13^C). ^a^ Chemical shifts may be interchangeable.

**Table 6S.** NMR data of Spiroindimicin B.

| **position** | **δ_H_ (J in Hz)** | **δ_C_** | **Cosy** | **HMBC** |
| --- | --- | --- | --- | --- |
| **1** | NH | - | - | - |
| **2** | 6.98 s | 111.7 | - | 5-3 |
| **3** | - | 127.9 | - | - |
| **4** | - | 142.6 | - | - |
| **5** | - | 117.4 | - | - |
| **6** | - | 162.2 | - | - |
| **7** | 3.59 s | 51.1 | - | 5-6 |
| **1’** | - | - | - | - |
| **2’** | 4.09 d (8.8)  3.67 d (8.8) | 65.2 | - | 10’-3’-4’-4-2’’-9’ |
| **3’** | - | 53.1 | - | - |
| **4’** | - | 135.8 | - | - |
| **5’** | 6.34 d (2.1) | 123.3 | 7.05 | 3’-7’-9’ |
| **6’** | - | 124.1 | - | - |
| **7’** | 7.04 (2.1, 8.5) | 128.6 | 6.34-6.64 | 5’-9’ |
| **8’** | 6.64 d (8.5) | 109.4 | 7.05 | 5’-4’ |
| **9’** | - | 153.8 | - | - |
| **10’** | 2.95 s | 36.3 | - | 2’-9’ |
| **1’’** | NH | - | - | - |
| **2’’** | - | 156.6 | - | - |
| **3’’** | - | 112.8 | - | - |
| **4’’** | - | 122.9 | - | - |
| **5’’** | 7.58 d (2.0) | 119.2 | 6.99 | 3’’-7’’-4’’ |
| **6’’** | - | 126.1 | - | 5’’-7’’-6’’-9’’ |
| **7’’** | 6.99 dd (2.0, 8.6) | 121.5 | 7.25-7.58 | 5’’-4’’-8’’ |
| **8’’** | 7.25 d (8.6) | 114.1 | 6.99 | 4’’-6’’ |
| **9’’** | - | 139.9 | - | - |

Sample was measured in MeOH. Data were recorded on Bruker Ascend 700 MHz. Spectra referenced to internal solvent for MeOH at 3.31 ppm (^1^H) and 49.0 ppm (^13^C).

**Table 7S.** NMR data of Spiroindimicin E.

| **position** | **δ_H_ (J in Hz)** | **δ_C_** | **HMBC** |
| --- | --- | --- | --- |
| **1** | NH | - | - |
| **2** | 6.95 s | 111.4 | 5-4 |
| **3** | - | 125.7 | - |
| **4** | - | 142.9 | - |
| **5** | - | 117.0 | - |
| **6** | - | 162.3 | - |
| **7** | 3.58 s | 52.1 | 6 |
| **1’** | - | - | - |
| **2’** | 4.09 d (8.8)  3.69 d (8.8) | 65.0 | 10’-7-4-9’ |
| **3’** | - | 52.8 | - |
| **4’** | - | 136.4 | - |
| **5’** | 6.34 d (2.0) | 123.7 | 3’-7’-9’ |
| **6’** | - | 123.1 | - |
| **7’** | 7.044 (6.06) | 128.1 | 5’-9’ |
| **8’** | 6.64 d (8.5) | 109.7 | 5’-4’ |
| **9’** | - | 153.7 | - |
| **10’** | 2.95 s | 36.2 | 2’-9’ |
| **1’’** | NH | - | - |
| **2’’** | - | 152.3 | - |
| **3’’** | - | 118.4 | - |
| **4’’** | - | 122.0 | - |
| **5’’** | 7.28 d (0.7, 7.3) | 113.5 | 7’’-4’’ |
| **6’’** | 7.03 (1.72) | 121.0 | 5’’-7’’-4’’-9’’-8’’ |
| **7’’** | 7.045 (0.81) | 120.5 | 5’’-4’’-8’’ |
| **8’’** | 7.58 (0.7, 7.4) | 119.7 | 5’’-4’’-9’’ |
| **9’’** | - | 141.7 | - |

Sample was measured in MeOH. Data were recorded on Bruker Ascend 700 MHz. Spectra referenced to internal solvent for MeOH at 3.31 ppm (^1^H) and 49.0 ppm (^13^C).

**Table 8S.** NMR data of Spiroindimicin F

| **position** | **δ_H_ (J in Hz)** | **δ_C_** | **Cosy** | **HMBC** |
| --- | --- | --- | --- | --- |
| **1** | 11.35 s | - | 7.09 | 5-3-4 |
| **2** | 7.09 (2.7) | 111.0 | 11.35 | 5-3-4 |
| **3** | - | 125.5 | - | - |
| **4** | - | 141.5 | - | - |
| **5** | - | 115.2 | - | - |
| **6** | - | 159.9 | - | - |
| **7** | 3.47 s | 50.1 | - | 6 |
| **1’** | - | - |  | - |
| **2’** | 3.64/3.90 d | 63.1 | - | 10’-3’-4’-4-9’-2’’ |
| **3’** | - | 51.4 | - | - |
| **4’** | - | 132.2 | - | - |
| **5’** | 6.33 d (7.3) | 121.6 | 6.44 | 7’-9’ |
| **6’** | 6.44 t (7.3) | 117.1 | 6.33-7.06 | 8’-7’-4’ |
| **7’** | 7.06 t (1.2, 8.1) | 127.6 | 6.44-6.71 | 8’-5’-4’-9’ |
| **8’** | 6.71 d (8.0) | 107.2 | 7.06 | 6’-4’ |
| **9’** | - | 153.3 | - | - |
| **10’** | 2.9 s | 35.7 | - | 2’-9’ |
| **1’’** | 11.4 s | - | - | 3’’-4’’-9’’-2’’ |
| **2’’** | - | 155.7 | - | - |
| **3’’** | - | 110.6 | - | - |
| **4’’** | - | 120.8 | - | - |
| **5’’** | 7.72 s | 117.7 | - | - |
| **6’’** | - | 123.1 | 6.99 | 4’’-8’’ |
| **7’’** | 6.99 dd (2.04, 8.6) | 119.7 | 7.25 | 5’’-8’’-9’’ |
| **8’’** | 7.25 (8.6) | 123.5 | 6.99-11.4 | 3’’-7’’-8’’-9’’ |
| **9’’** | - | 138.0 | - | - |

Sample was measured in MeOH. Data were recorded on Bruker Ascend 700 MHz. Spectra referenced to internal solvent for MeOH at 3.31 ppm (^1^H) and 49.0 ppm (^13^C).

**Table 9S.** Minimal inhibitory concentrations (μg/ml [μM]) of isolated compounds against bacterial test cultures.

| Organism | lycogarubin C | lynamicin E | spiroindimicin B | spiroindimicin E |
| --- | --- | --- | --- | --- |
| *Bacillus subtilis* | >100 [>240] | >100 [>223] | 25 [57] | >100 [>247] |
| *Escherichia coli* | >100 [>240] | >100 [>223] | >100 [>228] | >100 [>247] |
| *Pseudomonas putida* | >100 [>240] | >100 [>223] | >100 [>228] | >100 [>247] |


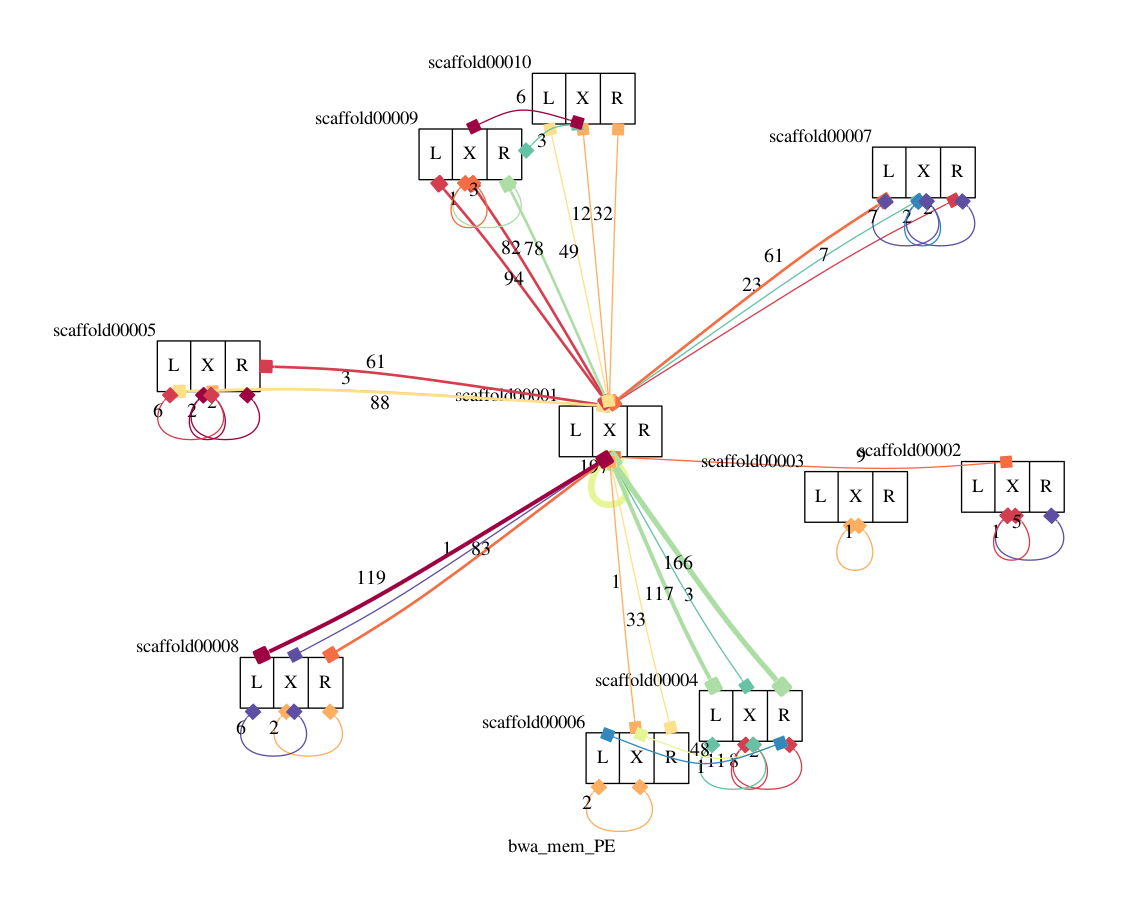


**Figure 1S.** Paired-end links between scaffolds: each link represents a read pair. L, left edge; R, right edge; X, neither left nor right edge (i.e. somewhere else in the scaffold). Mean PE insert size is 470 with standard deviation ~200; thus scaffold edge was estimated at 670bp. Loops depict self-links. Line weight is proportional (within fixed min/max limits) to the number of links, which is also shown next to each connecting line. Scaffold 1 (in the center) has no PE links from its L/R edges.


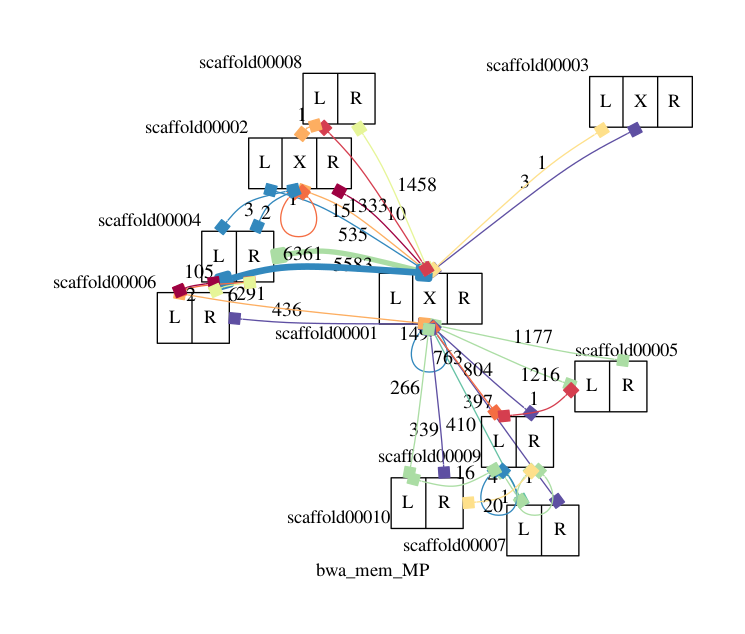


**Figure 2S.** Mate-pair links between scaffolds: each link represents a read pair. Mean MP insert size is 8100 with standard deviation ~1000; thus scaffold edge was estimated at 9100bp. Scaffold 1 (in the center) has no MP links from its L/R edges.

RT: 9.54 min

m/z = 414.1485


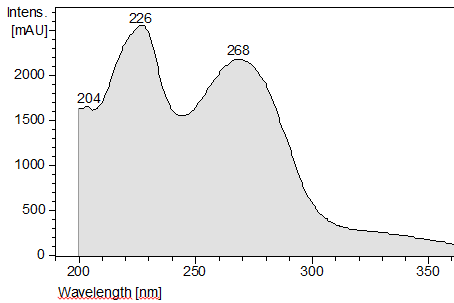

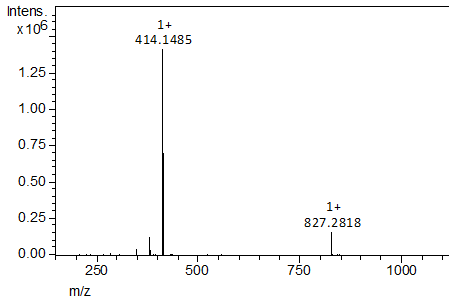


**Figure 3S**. MS chromatogram of lycogarubin C


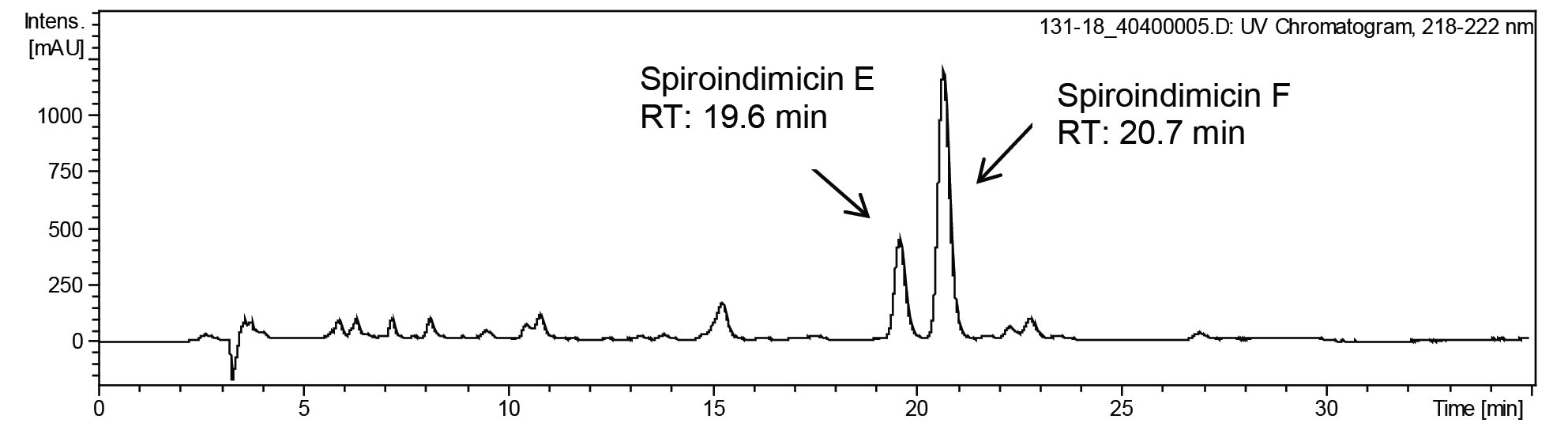


Spiroindimicin E Spiroindimicin F


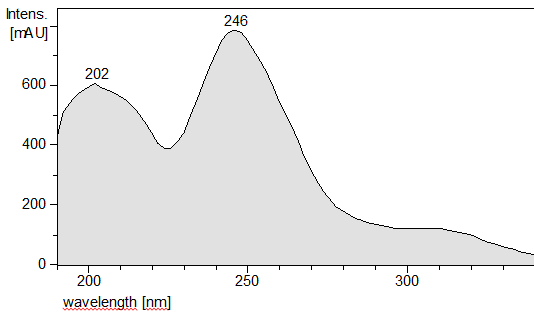

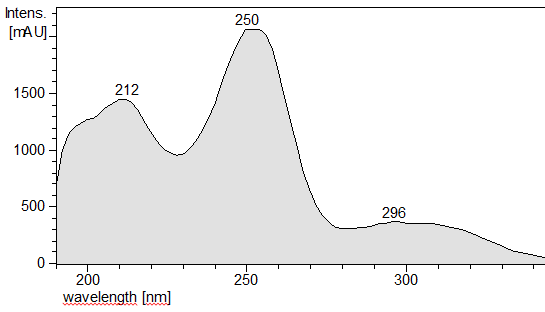


**Figure 4S.** Semipreparative HPLC chromatogram for the isolation of spiroindimicin E and F.


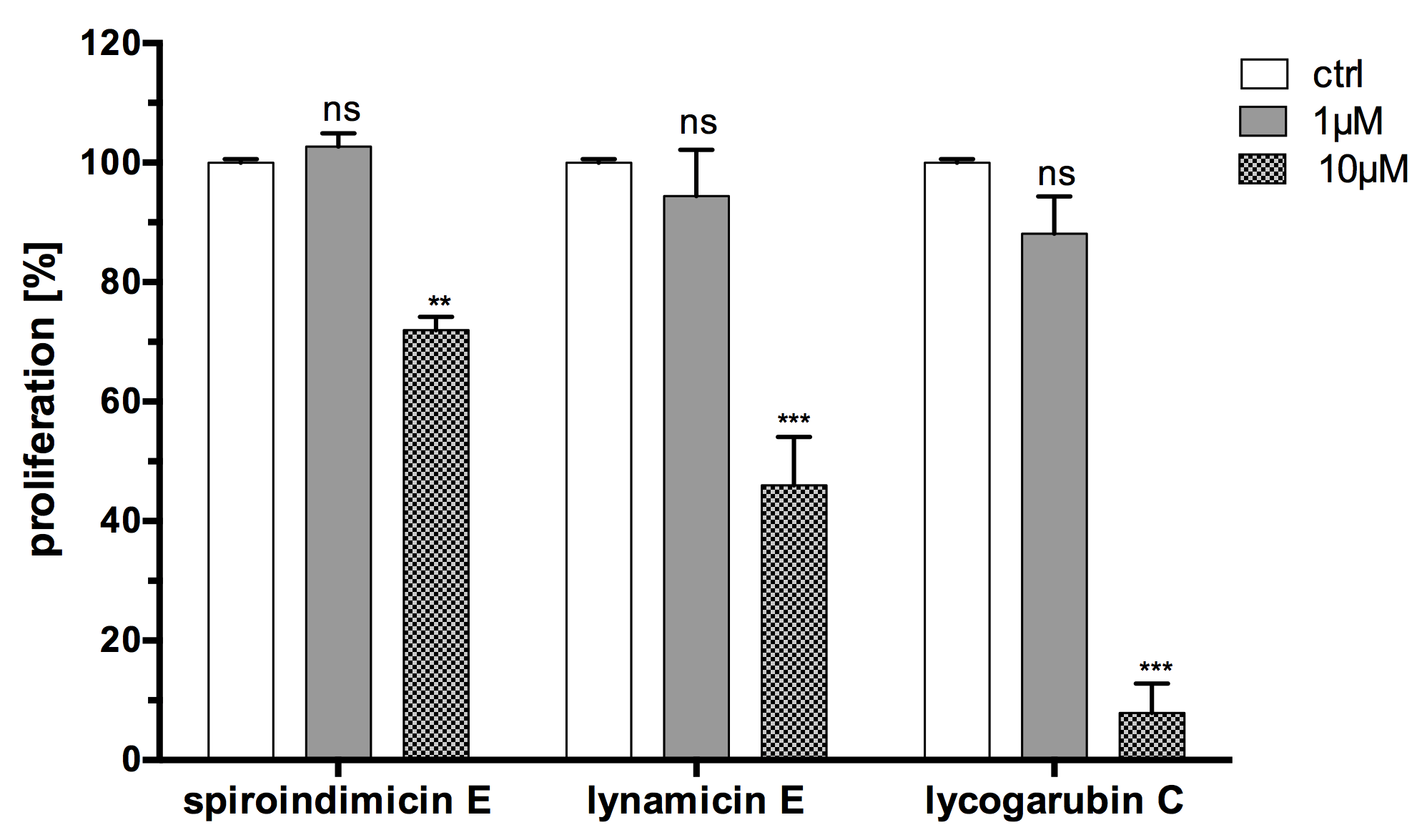


**Figure 5S.** **Growth-inhibitory effects on T24 bladder carcinoma cells.** Cancer cells were treated for 72h with increasing concentrations of the indicated substances and proliferation rate was analysed by staining with crystal violet.


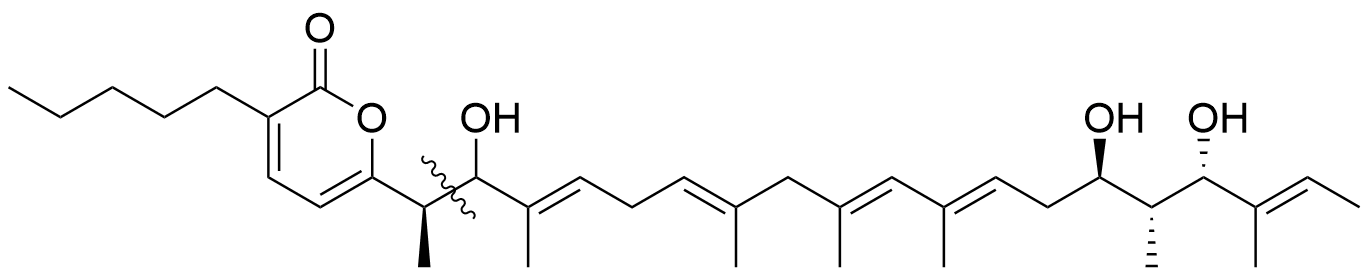


321.34 (without 3x water)

Lagunapyrone A

MS

MS2

MS

MS2

Lagunapyrone B

MS

MS2

Lagunapyrone C

MS

Lagunapyrone D

MS

Lagunapyrone E

MS

**Figure 6S.** MS2 for lagunapyrones (continuation).

**Figure 7S**. Proposed biosynthesis pathway for lynamicins and spiroindimicins.
